# Barriers to Accessing Primary Dental Care in Adults with Alcohol Dependence: A Qualitative Study

**DOI:** 10.1177/23800844231169642

**Published:** 2024-01-27

**Authors:** C. Bowes, M. Breckons, R.D. Holmes, J. Durham, B.K. Bareham

**Affiliations:** 1 School of Dental Sciences, Newcastle University, Newcastle, UK; 2 NIHR In-Practice Fellowship, Newcastle, UK; 3 Population Health Sciences Institute, Newcastle University, Newcastle, UK; 4 Applied Research Collaboration North East/North Cumbria, Newcastle, UK

**Keywords:** health inequities, alcoholism, facial pain, toothache

## Abstract

**Background:**

People with alcohol dependence (AD) frequently experience oral health problems, but their dental attendance is poor, with limited evidence to the reasons why from their perspective.

**Objective:**

To explore perceived barriers, motivators, and facilitators to accessing primary dental care in people with AD.

**Methods:**

Qualitative study consisting of remote one-to-one and group semistructured interviews with a convenience sample of adults with lived experience of AD in northern England. Data were audio-recorded, transcribed, and coded. A reflexive thematic analysis method was used; use of COM-B model informed data interpretation.

**Results:**

Twenty adults with lived experience of AD participated in 18 one-to-one interviews and 1 group interview (of 3 participants). Barriers to access were fear and physical, social, and environmental factors (physical effects of AD, financial barriers, nonprioritization of oral health). Motivators to access were pain and prioritization of oral health. Facilitators to access were patterns of alcohol use (i.e., sobriety) and dental service provision within recovery services.

**Conclusions:**

Fear of “the dentist” is a major barrier to accessing dental care, and pain is the primary motivator, among people with AD, although neither are unique to this population. Fear and physical, social, and environmental barriers to access contribute to problem-oriented attendance, which negatively affect oral health outcomes. Opportunity to facilitate attendance increases when a person is in remission from AD through their physical capabilities improving. Increasing capability and opportunity can influence attendance beyond the automatic motivation of pain. Provision of dental care within recovery services could facilitate access to care. Understanding the “web of causation” is key to developing any intervention to improve dental access in people with AD. Further research is needed from the perspective of other adult populations with lived experience of AD, as well as of dental professionals, to gain deeper insight into barriers, facilitators, and possible solutions.

**Knowledge Transfer Statement:**

*The results of this study can help dental professionals understand factors affecting access to primary care in people with alcohol dependence to provide knowledge that may reduce stigma surrounding the disease. Results also demonstrate areas for intervention development for public policy.*

## Background

Alcohol dependence (AD) is a chronic and progressive psychological illness characterized by an inner drive to use alcohol despite associated physical, mental, and/or social harm and a withdrawal state when use is stopped (World Health Organization [WHO] 2018). In England, around 1 in 100 of the adult population is alcohol dependent (Public Health England 2017a).

Harmful alcohol use is a major public health problem. Globally, alcohol use is attributable to 3.3 million deaths per year, and alcohol use at dependent levels is causatively linked to over 200 health conditions (WHO 2018). AD is a significant risk factor for oral diseases, including dental caries, tooth wear, periodontal disease, and oral cancer (Priyanka et al. 2017). The acidic nature of alcohol lowers the pH in the oral environment, which increases the risk of caries and erosive tooth surface loss. Risks are amplified further by xerostomia, an additional consequence of AD. Smoking prevalence is high among people living with AD, as unhealthy behaviors often co-occur (Meader et al. 2016). Risk of periodontal disease and oral cancer is therefore multiplied in this population (O’Sullivan 2011). Furthermore, people in recovery from AD often increase their sugar consumption in early stages of remission; this is due to craving sugar in lieu of alcohol cravings (Jeynes and Gibson 2017) or trying to address a pleasure–reward imbalance following alcohol abstinence (Stickel et al. 2016). Increased sugar consumption heightens caries risk in recovery.

People with substance use disorders (SUDs), including AD, have low uptake of dental care (Cuberos et al. 2020), with attendance being prompted by urgent, painful conditions (Sheridan et al. 2001; Robinson et al. 2005). This leads to preventable, treatable dental conditions, becoming more severe or life-threatening due to their sequelae, especially if associated with immunosuppression resulting from AD. There is a paucity of evidence regarding dental attendance of people with AD specifically, with literature more often focusing on attendance of people with opioid-related SUDs.

This study aims to explore barriers, facilitators, and motivators to accessing dental care for people with AD using qualitative methods to gain an in-depth understanding from the perspective of people with lived experience of this condition.

## Method

### Ethical Approval

This study was approved by Newcastle University’s Faculty of Medical Sciences Research Ethics Committee (Ref. 2051/8989).

### Design and Sample

This qualitative study used semistructured interviews to explore the perceived barriers, motivators, and facilitators to accessing primary dental care among adults with lived experience of AD. Eligibility criteria included ≥18 years old living in the United Kingdom, experience of AD, family member/carer of someone with AD, and ability to converse in and understand English.

Convenience sampling, with snowballing, was employed. The study was advertised via Humankind (third-sector alcohol service) on their social media platforms and flyers distributed to service users. Prospective participants contacted me (the lead researcher C. B) via phone or email to express interest in participation. Patient information sheets, a screening questionnaire (to confirm participants met the eligibility criteria), and consent forms were posted (with a return envelope) or accessed electronically. The screening questionnaire and consent form were required to be completed before participation.

### Data Collection

As the lead author (C.F.B.), I conducted 18 one-to-one interviews and 1 group interview of 3 participants between July and October 2021. Two participants were family members of people with AD. One person participated in both the one-to-one and group interview. Data were collected remotely via telephone/video call due to COVID-19 restrictions. Interviews followed a structured topic guide, designed by C.F.B. with input from R.D.H., J.D., and 1 participant (male participant 13) who had previously participated in the project “Drink Wise, Age Well” and therefore had prior experience of research participation. The topic guide was reflexively modified throughout data collection to explore emerging concepts. Interviews were audio-recorded, professionally transcribed verbatim, and anonymized. C.F.B. cross-checked transcripts with audio-recordings for accuracy. R.D.H. checked the first transcript to aid with reflexive modification of the topic guide. Data collection stopped when theoretical sufficiency was achieved, whereby I judged that additional data would provide little further insight.

### Data Analysis

Qualitative data analysis was guided by a reflexive thematic analysis approach (Braun and Clarke 2019). This methodology enables conceptualization of themes from patterns within the data and, in line with a critical realist paradigm, acknowledges the importance of researcher subjectivity as a resource and not an antithesis to the generation of knowledge (Fletcher 2017; Braun and Clarke 2021). In accordance with reflexive thematic analysis and the critical realist paradigm underpinning this body of work, as the lead author, I acknowledge that I have an active role in constructing generated data and the findings presented.

A critical realist paradigm enables critical engagement with the perspectives of people with lived experience of alcohol dependence and use existing theory. At the same time, it is acknowledged that my empirical reality will differ from that of the research participants, which ultimately serves to provide a richness in data analysis and interpretation through my reflexivity and enables consideration of possible future interventions.

My perspective of this topic, as a dentist, contributed to the development of the research question and chosen methodological approach to the study (collection, analysis, and interpretation, with input from all authors). As such, I required reflexivity to examine my positionality (as a dentist who drinks alcohol but with no lived experience of AD, in a reality where dependence is stigmatized) in relation to how this may influence data generation. This process of reflexivity has allowed me to be transparent in my methods and has plainly stated that my understanding of reality does influence data generation, but this is not in conflict with the methodology of reflexive thematic analysis or my critical realist position.

Microsoft Excel (Microsoft Corp.) was used to manage data. Phase 1 of data analysis was immersion in the data set, through active recursive reading of transcripts, which allowed for familiarization with the data and writing familiarization codes. Phase 2 involved inductively generating initial codes relating to patterns within the data, enabling coding of the whole data set; generated codes were numbered. R.D.H. and J.D. provided input in order to sense-check data coding. Each transcript was coded using this developing coding framework, with the addition of new codes where required. The coding framework was refined, removing duplicates/similar codes and those that were less important to the research aims and objectives or were at a descriptive rather than conceptual level. The process was repeated with the fully developed coding framework (when no new codes were identified in the data). Three a priori categories were used to aid organization and reflection of coded data in relation to the research question: 1) barriers, 2) facilitators, and 3) motivators (to dental attendance). Codes were examined to consider the category they belonged to.

In phase 3, categorized codes were collated into initial explanatory themes in relation to the data and my critical realist positioning. For example, “fear of the dentist” was a theme developed inductively both from the data and from my experiences as a dentist, where patients commonly tell me that “I hate ‘the dentist.’” Phase 4 included development and review of themes by reorganizing data within initial explanatory themes and ensuring themes accurately captured the essence of the data (C.F.B., M.B., B.K.B.); themes are patterns of shared meaning in the data that center on a focal concept (Braun and Clarke 2021). In line with a worked example of reflexive thematic analysis (Byrne 2022), themes selected were distinctive from each other and collectively, as a thematic framework, felt to capture all of the pertinent issues described by participants relating to the research aims.

Input from M.B. and B.K.B. was sought in phase 5 in order to sense-check whether themes were appropriately refined, defined, and named in relation to the data, so that an outsider could make sense of the data presented. Phase 6 involved writing the research report.

The COM-B model (Michie et al. 2011) informed data interpretation through mapping of constructs and themes. In line with critical realism, the COM-B model provided a framework to understand reality in a way that helps identity the causal mechanisms of phenomena at the empirical level. This framework offers a helpful way to interpret dental access behavior for people with AD in relation to 3 components: capability (individual psychological and physical capacity to attend), opportunity (influences by external physical and social factors), and motivation (automatic and reflective drive) (Michie et al. 2011). In line with the objectives of this research, it was important to consider data in relation to the COM-B model as it enabled identification of behavioral factors that could be targeted in an intervention.

## Results

Interviews lasted between 14 and 60 min. Participant ages ranged between 22 and 68 y; *n* = 11 (over half of the sample) identified as being of female gender. All participants were White British and reflected a range of socioeconomic backgrounds. Six participants (*n =* 3 male) reported previous illicit SUDs prior to or during AD. Participant demographics are provided in Table 1.

**Table 1.** Demographics of Participants.

Characteristic	*n*
Sample size (female)	20 (11)
Age, y	
20–29	4 (3)
30–39	2 (0)
40–49	8 (7)
50–59	4 (0)
60+	2 (2)
Ethnicity	
White British	20
Participation	
One-on-one interview	17 (10)
Group interview One-on-one and group	2 (1) 1 (0)
Experience of alcohol dependence Direct Indirect	18 (10) 2 (1)

Each participant was assigned a study ID, through random number generation (between 1 and 20) for anonymity. Quotes provided reflect common sentiments and illustrate themes most effectively. Themes are presented in Table 2 for clarity. Developed themes are mapped to COM-B in Table 3.

**Table 2.** Themes and Subthemes by Category for Illustrative Purposes.

Category	Theme	Subtheme
Barriers	Fear	Fear of the dentist Fear of stigmatization and discrimination
	Physical, social, and environmental factors	
Motivators	Pain	
	Prioritization of oral health	
Facilitators	Patterns of alcohol use	
	Integration of dental provision within recovery services	

**Table 3.** Themes Identified within This Study Categorized into Components of the COM-B Model (Michie et al. 2011).

COM-B Component	Theme	Relevance
Capability		
Physical (possessing physical skills, strength, or stamina)	Physical, social, and environmental factors	Physical side effects of AD (i.e., being unwell, lacking capacity as a barrier to attendance)
	Patterns of alcohol use facilitating attendance	Alcohol use preventing withdrawal symptoms enabling attendance
Psychological (possessing knowledge, psychological skills, strength, or stamina)	Prioritization of oral health	Awareness of benefits of prioritizing dental health, including accessing dental care
Opportunity		
Physical (what the environment allows/facilitates through time, triggers, resources, locations, physical barriers, etc.)	Physical, social, and environmental factors	Financial constraints affecting affordability of dental care Difficulty accessing NHS dental care (due to relocation; COVID-19)
	Integration of dental provision within recovery service	AD-specific dental provision within recovery services could create opportunity to facilitate access
Social (what the environment allows/facilitates through interpersonal influences, social cues, and cultural norms)	Fear of stigmatization	Improving awareness of AD as a disease may reduce fear of stigmatization from public and dental professionals within dental settings (altering social norms)
	Integration of dental provision within recovery service	Receipt of dental care by peers from similar substance use background improving social norms
Motivation		
Automatic (emotions, wants, needs, desires, impulses, and reflex responses)	Fear	Fear of dental treatment and stigmatization from dental professionals
	Pain	Pain as a reflexive motivator is independent of alcohol use
	Prioritization of oral health	Dental appearance and function as a motivator for dental attendance
Reflective (self-conscious planning and evaluating “good” vs. “bad”)	Physical, social, and environmental factors	Beliefs around affordability of dental care
	Prioritization of oral health	Wanting to influence child’s oral health and relationship with dentist Awareness of the importance of dental attendance

AD, alcohol dependence; NHS, National Health Service.

### Category 1: Barriers to Attendance

This category comprised 2 themes: fear and physical, social, and environmental factors.

#### Theme: Fear

##### Subtheme: Fear of “the dentist”

The feeling of fear relating to dental attendance was evident across the majority of interviews. Fear of “the dentist” was described as a barrier to attendance both when actively dependent and when in remission. Some participants felt that using alcohol prior to an appointment alleviated fear, although AD itself appeared to be independent of the fear experience: many participants also reported fearing “the dentist” prior to being diagnosed with AD. Fear was reported by both men and women across age ranges.

For some participants, fear of “the dentist” stemmed from 1 traumatic dental experience during childhood:

It’s [going to the dentist] just scary . . . I had to go to the dental hospital when I was a kid . . . they put me to sleep, I had to have six teeth taken out and six fillings and that, but just before I went for surgery another kid came back from his [surgery]. I was like nah [no] and then I woke up. I didn’t know where the fuck I was. (25-year-old female, participant 6)

Other participants described a more generalized fear of the dentist, although still stemming from childhood:

I’ve always been petrified, even like from a young age, I just I hate the sound of the drill, I hate the feeling in my mouth, I feel like I can’t breathe whenever they’re doing anything. (42-year-old female, participant 4)

Some participants described fear in anticipation of attending dental appointments. One participant feared the noises and pain associated with attendance:

It’s just the thought of going to the dentist. Once you’re in the dentist chair I’d be terrified of all this [makes drill noise] into your nerves. Do you know what I mean? I’m still scared of all that like. (57-year-old male, participant 20)

Another participant, who was related to someone actively dependent on alcohol, described a nonspecific anticipatory fear experienced by her sister when actively dependent on alcohol:

She’s scared of the dentist. She’s OK when she gets there, it’s just getting there. She’s up all night. There’s always drama when she gets in . . . once she’s in she’s reassured. It’s just her getting there in general. She tried to put it off and off. (47-year-old female, participant 10)

##### Subtheme: Fear of stigmatization and/or discrimination

Fear of stigmatization and/or discrimination was typically experienced when actively dependent. Some participants felt that now they were in remission, they had come to consider AD as a disease rather than a flaw and were therefore less ashamed, with reduced fear of stigmatization and discrimination. When actively dependent, participants reported that they feared being judged by dental professionals, support staff, and other patients for their AD; this demotivated them from attending:

Yeah, being judged I think like there’s a really bad stigma still against alcoholics, like you’re just branded as a certain category of person that you might as well be sitting on the street with a bottle wrapped in a paper bag. (42-year-old female, participant 4)

Many described fear of discrimination that they might be refused treatment if they had used alcohol prior to an appointment and avoided attending to prevent embarrassment. One participant described avoiding visiting the dentist unless she was in one of her “sober phases”:

I wouldn’t go to the dentist when I was drinking . . . that just wouldn’t happen . . . I knew they wouldn’t remove it [wisdom tooth] if I was drunk ’cos they couldn’t give us [me] the anesthetic. (29-year-old female, participant 1)

Other participants reported attending when actively alcohol dependent but tried to mask the smell of alcohol to hide their dependence and avoid stigmatization and/or discrimination. One participant described an almost ritualistic approach to masking the smell:

If I had a can in the morning and the appointment was not ’til 3 o’clock I’d just [do the] usual stuff: brush my teeth; Listerine; chewing gum, because the one can maybe just kept you straightened out until later on ’til once you got out the dentist . . . and then you would have a drink when you come out. (ID.66/M_56, 56 year old female, participant 18)

Another participant described their approach to masking the smell of alcohol:

I was trying to scrub myself down, clean my mouth out so I didn’t smell of alcohol. (60-year-old male, participant 12)

Participants feared discrimination from dental professionals for the condition of their teeth as they felt that dentists would judge them for both being alcohol dependent and being responsible for their poor oral health as a consequence of dependence. This led to participants feeling culpable and therefore ashamed of their poor oral health. When asked why they felt ashamed, 1 participant replied,

I shouldn’t be ashamed of it [AD] as much as I am. I shouldn’t be ashamed of it, but I am ashamed of it . . . I think it’s probably because of the stigma that’s attached to being an alcoholic . . . I just thought it couldn’t be me because I wasn’t that kind of person but it’s anybody. It’s just the idea I suppose it’s the denial of being an addict, you just don’t want to associate yourself with those people, but I am an alcoholic at the end of the day. I can’t undo it. I just have to live with it. (42-year-old female, participant 5)

The feeling of shame is exemplified by 1 participant who described what they would say to themselves if they were a dentist:

What the fuck mate? Look at the fucking state of them [teeth]. That’s probably what I’d think if I was a dentist. You know it’s just almost like, “make an effort mate.” (57-year-old male, participant 20)

Although a common fear, only 1 participant described experiencing discrimination while visiting the dentist. She reported that she had attended for an emergency appointment complaining of “chronic pain,” but the dentist was a “a bit dismissive” and sent her away with some sensitive toothpaste; it is unclear if the dentist was disbelieving of her pain and/or unwilling to prescribe painkillers. It was not until she reattended, having gone to her general practitioner who prescribed antibiotics for a dental abscess, that the dentist treated (removed) the tooth. In relation to this incident, the participant said,

I do feel very self-conscious when I go there, or I did when I was drinking. I wondered whether [my AD] might have had an impact at the time and he thought “this person likes a drink and I’m not going to take it as seriously” as it were with the pain that I was describing. (55-year-old female, participant 9)

#### Theme: Physical, social, and environmental factors

This theme discusses individual physical barriers to attendance experienced by participants, societal factors impacting attendance, and physical environmental factors as a barrier to attendance.

Physical symptoms associated with AD, such as feeling sick and tired, were a barrier to visiting the dentist, as individuals felt too unwell to attend appointments:

Sometimes if I wasn’t feeling well through the drink or whatever I would cancel. (40-year-old female, participant 7)

One participant (family member of a person with AD) reported that their sibling required an escort to attend with them, due to “going blind” as a result of AD. This created a barrier to attendance due to loss of their independent physical capabilities:

She wouldn’t go [to the dentist] on her own. She can’t see from the alcohol. She can’t see properly . . . she has to have someone with her . . . she’s not independent enough. (47-year-old female, participant 10)

For many people with active AD, motivation to attend was depleted, as their competing motivation was the “next drink.” The “next drink” was most often prioritized over attending dental appointments when actively dependent:

I don’t think I went [to the dentist] at all in them two years. I just couldn’t be bothered. All you’re really bothered [about] is just going and getting my first bottle. (42-year-old female, participant 8)

Several participants reported that alcohol helped manage their dental pain, mitigating any perceived need to access dental care:

[I had] pericoronitis and yeah, that was very painful, and alcohol definitely numbed that. (30-year-old male, participant 17)

I’ve always found like drinking does take like physical pain away. (22-year-old female, participant 2)

One participant described having toothache at work, and drinking alcohol to suppress pain was opted for over seeking care. When asked how he would manage the pain, he said,

As soon as I finished work, I’d just go straight to the drinks because—and it was an old dentist who once told me this—“Have a couple of straight whiskeys, right? But gargle with it, right? And whether you want to spit it out or not is up to you, but whiskey helps clear the infection.” But I said, “I’m not spitting it out. I’ll fucking drink the fucker.” (56-year-old male, participant 18)

Financial cost of accessing dental care was a frequently reported barrier. Participants often had financial difficulties linked to job loss and homelessness, which were common consequences of AD among this sample and could persist into recovery. As such, participants felt they lacked opportunity to attend as they perceived the cost of dental treatment was beyond their means. Participants felt paying for dental treatment was not a priority due to other competing motivators such as buying alcohol (when actively dependent) or buying food (now that in remission):

I literally don’t have the money to pay for it so I can’t. (40-year-old female, participant 7)

Relocation was also common among participants as a result of job loss, homelessness, and attending residential rehab due to AD. Participants reported that they had lost jobs due to turning up to work drunk or missing work due to intoxication or hangovers; job loss could subsequently lead to homelessness for some. Relocation created a barrier to access, as a new dentist had to be sought. This could be difficult in a climate with a shortage of National Health Service (NHS) dentists and waiting lists for new patients:

I was made homeless eventually throughout lockdown, so I moved to a different part of [the country]. I went to try and get a local dentist, but there was, like, a year waiting list, um, just for NHS patients. (32-year-old male, participant 15)

The ongoing COVID-19 pandemic, with its additional impact on access to dental care through prioritization of urgent care of registered patients, had also affected participants’ dental attendance. This, together with self-stigmatizing attitudes of not being deserving of dental care due to perceived culpability for poor oral health (reported earlier), reduced motivation:

I don’t feel like I want to pressure my local dentist to get me back in, particularly when there’ll be probably people who are suffering. (50-year-old male, participant 13)

### Category 2: Motivators to Attendance

This category consisted of 2 themes: theme 3 (pain) and theme 4 (prioritization of oral health).

#### Theme: Pain

Most participants described pain as the primary motivator to attendance when alcohol dependent:

Nothing would make me go the dentist when alcohol dependent except pain. (42-year-old female, participant 8)

I only go to the dentist when I’m in pain. Like I literally avoid the place. (25-year-old female, participant 6)

For some, pain had to become intolerable before they would seek dental care:

I would wait until I was in that much pain that I would go, even though I would have pain before it would have to get to a certain intensity before I would actually go. (42-year-old female, participant 3)

Toothache persisted to be the main motivator to attendance for some participants when in recovery. One participant described toothache as being worse than other causes of pain and therefore would seek care:

Well, I’ve got a high—quite a high threshold with pain, right? I have and I’m, I’m not giving the big one here . . . but when it comes to toothache, I’d always go for nine—nine or ten out of ten—because toothache is always excruciating. You can have a niggle, and then you might notice it goes away in a couple of days but, but toothache is just—toothache is excruciating. (56-year-old male, participant 18)

Among participants in remission, 1 participant described how toothache motivated attendance to resolve their pain due to fearing further addiction if they took painkillers instead, recognizing their disposition to addiction through previous AD:

You know, you read all the stuff about opiates, people become addicted to these things, and I’ve had enough addiction already in my life. So, I thought I better get this dealt with. So, I’m probably very much more likely to go and see a dentist straight away and avoid having to take [painkillers]. (60-year-old male, participant 12)

#### Theme: Prioritization of oral health

Several participants described valuing and promoting their oral health when actively dependent, even if this did not motivate attendance:

I didn’t really go to the dentist much, for me, it’s like a once every couple of years thing that I do, and it was really low on my list of priorities when I was in active addiction but the way that I tried to counter act that was to try and take care of my teeth as much as I could. (30-year-old male, participant 17)

Appearance of teeth motivated attendance for 1 female participant, although this related to a significant instance where their front tooth was damaged due to alcohol-related trauma, rather than regular attendance to maintain their oral health:

I was absolutely mortal [very drunk], and I fell on a marble table and smashed my front tooth . . . it was like half me front tooth . . . I was like I’m not going to work; this has to get sorted, but it’s more vain I think—it looked awful. (42-year-old-female, participant 4)

Entering remission was a clear delineation of when oral health was “re”-prioritized. Maintaining oral health motivated attendance mainly when participants entered remission from AD. Oral health priorities included retaining and maintaining their natural teeth. When asked what their motivation for attendance was, they replied,

Saving me bottom teeth—I never want to have a bottom denture. (41-year-old female, participant 11)

Male and female participants equally felt that improving their oral health, including seeking dental treatment, could benefit their self-esteem when entering remission:

If I were to get my teeth sorted aye, it would give me massive confidence . . . yeah it would be almost beginning of the end of dependency. (57-year-old male, participant 20)

I’m very self-conscious when I talk, I’m very self-conscious, that’s why I’ve asked for the false ones and that because, you know, now I’m looking after myself again; you don’t realize when you’re drunk all the time. (42-year-old female, participant 3)

Two female participants, who were single mothers, reported that now they were in remission from AD as they wanted to positively influence their children’s oral health and attitudes to dental attendance. One female reported that their child had been removed from her by social services as a consequence of AD (but returned to her now in remission); the other reported that she “nearly lost her son” as a consequence of AD. These participants wanted to prevent their child experiencing dental fear like they had and were therefore motivated to attend themselves:

I just think [going to the dentist] is a good thing, you know, show my daughter as well you know, she loves going to the dentist. (40-year-old female, participant 7)

’Cos [because] I know I need to [go to the dentist] and I know that alcohol’s obviously not been great for me teeth, and I’ve got a little boy and I want to—I don’t want him to be scared, so I take him when I go. (42-year-old female, participant 4)

The subject of children was not brought up by participants in any interviews with male participants.

### Category 3: Facilitators to Attendance

#### Theme: Patterns of alcohol use

Most participants reported using alcohol immediately prior to a dental appointment while dependent. For some, alcohol helped overcome the previously mentioned anxiety-related barriers to dental attendance such as fear of “the dentist” or fear of stigmatization and/or discrimination.

Alcohol makes you stop worrying about everything; so, it takes away that anxiety about my teeth. (25-year-old male, participant 19)

Others reported that their alcohol use prior to dental attendance was necessary to feel “normal” by preventing withdrawal and associated symptoms, providing the physical capabilities to attend:

I used to drink vodka, um, every morning just to feel “normal,” um, for the benefit of the tape, I’m doing the ear rabbits quote things [laughs] . . . yeah just to feel, like, human. I mean to stop throwing up, essentially. (50-year-old male, participant 13)

She [my sister] has to have it [alcohol] before she gets out of bed. She can’t function without it. (47-year-old female, participant 10)

Participants reported that being in remission from AD facilitated dental attendance. Sobriety meant participants had the mental and physical capability to look after their oral health, including maintaining an oral hygiene regime and making and attending dental appointments:

I find that my life is much more manageable now that I’m not drinking so, yeah, I would probably have the time and the capacity to make appointments and things like that, whereas when I was drinking I could not. (30-year-old male, participant 17)

#### Theme: Integration of dental provision within recovery services

Due to the busyness of life in recovery, such as attending recovery meetings and occupying oneself to distract from alcohol cravings, providing an accessible service was suggested by participants as an opportunity to support attendance:

You haven’t got to go about it yourself like phoning up, booking it if it was sort of, I don’t know, there on a plate for you it’s there to take advantage of. (49-year-old male, participant 16)

Many participants suggested addiction-specific dental provision should be encompassed within recovery services, as they felt it would reduce stigma if a professional had greater understanding of AD:

There’s housing people who will work with you on your housing issues, and there’s people working with your family, but we just don’t have any sort of natural outlet to dental care where somebody could go. (60-year-old male, participant 13)

If participants felt that there was less stigma, then this would increase patient–professional trust:

I think someone [dentist] who was trained and knew about people in recovery and what they’re going through . . . would be really helpful. People would be more likely to be open with them I think. (55-year-old female, participant 9)

Participants reported that receiving appointment reminders would facilitate attendance as many participants reported poor memory as a long-term consequence of AD:

My memory is appalling . . . it’s absolutely shocking. You know when you were kids, and your mam would say “you’ll pickle your brain.” It [alcohol] does; it pickles your brain. (42-year-old female, participant 5)

Others reported that appointment reminders would provide written motivation to attend:

If I had, like, reminders to book appointments because I do stick to like when I’ve got an appointment I will stick to it, it’s just the taking my own initiative to kind of do it really. (22-year-old female, participant 2)

Participants emphasized the positive influence dental care could have on their journey into recovery, with the following participants describing how entering recovery is a new beginning, with a potential for being held back if dental health persists to be poor:

I think a dentist would be in-between the hard-core recovery services and the soft. Like I think kind of you’re just getting back into life and rehabilitation and getting back into living—it would be kind of a little bridge if you like. A dentist could be a really useful bridge. (57-year-old male, participant 20)

Well, you’ve just started a new life [when entering recovery] and if you’ve got [bad] teeth, rotten teeth or missing teeth, or anything like that, I think it makes the process [of recovery] really difficult. So, I think having the aid of a dentist could be really beneficial. (32-year-old male, participant 15)

## Discussion

This qualitative study is the first to our knowledge to provide rich and in-depth insights into the factors determining access to dental care among people with AD, from the perspective of this high-risk and low-attendance group. Barriers to attendance identified in this qualitative study included fear and physical, social, and environmental factors, such as financial barriers and illness as a consequence of AD. Motivators for attendance included experiences of pain (whether actively dependent or in remission) and prioritization of oral health (mainly when participants enter remission). Patterns of alcohol use (such as use of alcohol to feel “normal” or periods of sobriety) and integration of dental provision within recovery services were identified as facilitators to attendance. Our in-depth account identified gender differences in motivations to attend: where women with children were keen to model positive dental hygiene through visiting the dentist. Modifiable psychological, physical, and environmental factors affecting access to dental care, including capability, opportunity, and motivation, were identified across our data supported by using COM-B (Michie et al. 2011) during data interpretation.

Dental fear was a common barrier to attending dental care reported by participants and was experienced when actively dependent on alcohol, in remission, and often preceding the development of AD. Fear of the dentist is known to be a variable construct of fear: in anticipation of dental visits, fear of pain, and losing control to the dentist (Johnson et al. 1990). Evidence suggests that fear can stem from 1) direct learning through experience of traumatic dental encounters, 2) vicarious learning through family experiences or how dentistry is portrayed in the media, or 3) endogenous genetic factors and/or personality traits (Beaton et al. 2014). While fear of “the dentist” is not uncommon among the general population, with just under half (49%) of UK adults reporting some level of dental anxiety (Humphris et al. 2013), fear has been shown to be experienced at increased levels in people living with AD compared to the general population (Cuberos et al. 2020), as dependent drinking can both cause, and be caused by, mental health problems (NICE 2011). This suggests a greater endogenous response in people with AD, which requires unique attention.

In addition, reported use of alcohol prior to dental attendance to mitigate fear of the dentist during dependence is fathomable, given the short-term anxiolytic effects of alcohol use (Silberman et al. 2010), but this finding has not been reported in other studies. Review articles have surmised that patient use of alcohol prior to an appointment could lead to dentists refusing treatment through concern surrounding patient capacity to consent (Murthy 2019). Our study found participants were concerned about being discriminated against by being refused dental treatment for smelling of alcohol and therefore attempted to mask the smell, which again is a unique finding to this study.

Our study also highlighted that fear of stigmatization/discrimination was common to both people with active AD and those in remission, perpetuated through self-stigmatization. This finding is unsurprising, given AD is a highly stigmatized disorder (Keyes et al. 2010).

Cost of dental treatment was a reported barrier for those with AD. Perceived inability to afford treatment is likely due to the impact of AD on financial security, as job loss and high expenditure on alcohol are common consequences associated with AD (Fountain et al. 2003). However, many participants described being in receipt of Universal Credit (UC: government financial support), which entitles the recipient to free dental treatment. Despite this, attendance was not motivated. Interestingly, a qualitative study in Norway found that the offer of free dental treatment for people with SUDs did not motivate their attendance (Hovden et al. 2020). Therefore, our study may highlight that other barriers to attendance reported (i.e., job loss, homelessness, and relocation), which contribute to a chaotic lifestyle, are of greater significance in AD, and people with AD require a greater motivation than free dental treatment to facilitate attendance.

COVID-19 was a reported barrier to attendance, unique to the present study (conducted in 2021), as the pandemic has affected access to dental care in the United Kingdom and worldwide (Carter et al. 2020). This was due to primary dental care services being advised to reduce and subsequently cease routine dental care and face-to-face contact, relying on patients receiving urgent-only treatment in urgent care hubs (Carter et al. 2020). This resulted in a backlog of patients and increased waiting times for appointments, with routine appointments, such as checkups, unprioritized (CQC 2021). This backlog has affected the capacity of dental practices to accept new NHS patients (CQC 2021), meaning urgent appointments with dentists may be more challenging to access for a person not currently registered with a practice (such as people with AD). It is difficult to tell, and unlikely to be fully understood, to what extent NHS dental service availability negatively affects people with AD independent of COVID-19 due to previous literature detailing barriers pre-2006 dental contact reform. As such, this may be an area for further research, although the aftermath of COVID-19 is likely to persist for some time.

Pain was the leading motivator to attendance during active AD and persisted to be the leading motivator when in remission. Dental pain is not uncommon, with almost 1 in 10 UK adults reporting current pain (White et al. 2012). One-third of the adult population (including many with AD) are categorized as problem-oriented attenders (Steele et al. 2011).

Importantly, prevalence of dental pain and its severe sequelae, such as sepsis, increases with irregular dental attendance, poor oral hygiene regime, dental anxiety, lower socioeconomic status, and smoking (White et al. 2012), risk factors with which many participants in this study identified as having mostly when actively dependent. Our study found that dental problems can be further exacerbated by a tendency to self-medicate for dental pain, as found in other SUDs, which suppresses the severity of oral pain, thereby delaying perceived need to access dental care (Shekarchizadeh et al. 2019).

Prioritization of oral health became a motivator to attendance for participants upon entering remission from AD. Oral health for participants meant aesthetic improvement of their smile and increased functionality (ability to eat by getting false teeth); this could not be achieved unless they accessed dental care. As with studies of people with SUDs, improved oral health equated to a recovery of social competence, confidence, and self-esteem (De Palma and Nordenram 2005), potentially through what has been described by another study as creation of a “non-addict identity” (Bowes et al. 2019).

This study uniquely found that female participants were motivated to access dental care in order to influence their children’s behavior. These participants reported that they wanted to prevent their children fearing the dentist like they do; however, there may also be a moral motivation in taking their children to the dentist in terms of what could be considered “good” parenting. For example, moral motivation may have been influenced through previous child social service involvement as described by these female participants.

Our findings indicate great complexity involved in promoting routine dental attendance among people with active AD, as they experience a suite of compounding and multilayered barriers affecting all precursors to this behavior (capability, opportunity, and motivation) at both individual and environmental levels. Particularly, a primary motivation centering on alcohol consumption led to deprioritization of oral health, acting as a competing behavior to attending dental care. This resulted in a tendency toward problem-oriented attendance among this group.

While this study indicates that there may be limited potential for an intervention to address problem-oriented attendance during *active* AD due to multiple, compounding barriers, it is important for dental professionals to use each opportunity when people with AD *do* attend and “Make Every Contact Count” (Public Health England 2016). This is an established initiative to promote healthy behavior among health and social service users across health and social care in the United Kingdom, which makes use of every health and social care interaction to encourage healthy behavior change. In the dental setting, health promotion for people with active AD could include signposting of relevant recovery organizations (Public Health England 2017b).

A number of these barriers to attendance appeared to be overcome through recovery from AD as motivation to drink alcohol decreased and physical and psychological capability to attend increased through having 1) the capacity to make and keep to dental appointments and 2) the physical well-being needed to access care. Sobriety provided the psychological capability to prioritize oral health, as participants recognized the importance of, for example, receiving a denture (or “false teeth”) to replace teeth missing and damaged through the impact of alcohol and neglect of oral health to their recovery and self-esteem. However, a number of barriers already described continue to affect access to dental care during recovery from, for example, fear of the dentist.

Data from our study suggest that integration of dental provision within a recovery service could provide both physical and social opportunity to attend. Providing a designated service may facilitate engagement with dental care as the physical barrier to seeking an NHS dentist would be removed, and dental provision could be normalized within the recovery pathway, as with existing support provided for job seekers and homeless populations engaged in recovery services. This would also promote social opportunity to attend, as fear of stigmatization may be reduced through having a designated service among peers, where people with AD feel dental professionals are understanding of people with AD (Åstrøm et al. 2021). This approach has shown promise in pilot studies for populations with other SUDs (Charnock et al. 2004; Hare et al. 2012) but has not yet been explored in populations with AD.

To further inform the development of future interventions to promote access to dental care among people with AD, it is important to consider other key stakeholders such as dental professionals, who provide dental treatment, to gain their perspectives in relation to factors affecting this population’s capability, opportunity, and motivation to attend. Developed initiatives must be sensitive to feasibility in the context of public policy, and the impact of current government austerity measures on NHS service delivery, including availability and remuneration of the dental workforce for this high-needs group (Robinson et al. 2005).

## Study Limitations

The sample for this study lacked ethnic diversity as all participants were White British. Although this reflects the majority of the population in northern England (ONS 2021), it is important to be aware of subgroups, such as minority ethnic groups, that may experience greater stigmatization through alcohol prohibition and other unique barriers, facilitators, and motivators to dental attendance (Keyes et al. 2010).

## Conclusion

Fear of “the dentist” is a major barrier to dental attendance among people with AD, which, although reflective of the general population, deserves unique attention due to constitutional vulnerabilities of people with AD. Fear of stigmatization is a modifiable barrier, which is common to both those with active AD and those in remission. Fear and physical, social, and environmental barriers to attendance contribute to problem-oriented attendance, which affect oral health outcomes. Our findings suggest that opportunity to facilitate attendance increases when a person is in remission from AD through their physical capabilities improving. Intervention design should be cognizant of this. Increasing capability and opportunity can influence attendance beyond the automatic motivation of pain. Provision of dental care within recovery services, which may address fear of stigmatization, could facilitate access to care. Understanding the “web of causation” is key to developing any intervention to improve dental attendance in people with AD as there is no single barrier to attendance acting in isolation.

## Author Contributions

C. Bowes, R.D. Holmes, and J. Durham substantially contributed to concept and design; C. Bowes contributed to acquisition of data; C. Bowes, R.D. Holmes, J. Durham, M. Breckons and B.K. Bareham contributed to analysis; C. Bowes, M. Breckons, and B.K. Bareham contributed to interpretation; all authors contributed to intepretation and drafted, critically reviewd and gave final manuscript approval.
